# Supplementary material for: NSCLC EGFR Mutation Prediction via Random Forest Model: A Clinical–CT–Radiomics Integration Approach
Source: Adv Respir Med. 2025 Sep 26;93(5):39. doi: 10.3390/arm93050039 (PMC12562246; doi:10.3390/arm93050039)
Supplement: Supplementary file 1 [file arm-93-00039-s001.zip › Supplementary Table S1.pdf]

Supplementary Table S1. Final optimized hyperparameters for each Random Forest model

Model	Features Used	n_estimators	max_depth	min_samples_split	min_samples_leaf
Model 1	Clinical and CT – Full Feature Set	500	12	4	2
Model 2	Clinical and CT – Selected Features Only	400	10	3	1
Model 3	Radiomics – All Extracted Features	800	15	5	2
Model 4	Radiomics – Filtered Key Features	600	14	4	2
Model 5	Combined – Selected CT, Clinical, and Radiomic Features	700	12	3	1

(Grid search ranges: n_estimators = 100–1000, max_depth = 3–20, min_samples_split = 2–10, min_samples_leaf = 1–5. AUC used as optimization metric.)
