# Supplementary material for: NSCLC EGFR Mutation Prediction via Random Forest Model: A Clinical–CT–Radiomics Integration Approach
Source: Adv Respir Med. 2025 Sep 26;93(5):39. doi: 10.3390/arm93050039 (PMC12562246; doi:10.3390/arm93050039)
Supplement: Supplementary file 1 [file arm-93-00039-s001.zip › Supplementary Table S2.pdf]

Supplementary Table S2. Confusion matrices of the five Random Forest models aggregated over stratified 5-fold cross-validation on the full cohort (EGFR-WT = 98; EGFR-Mutant = 40). Rows = true classes ; Columns = predicted classes.

Model	True Class	Predicted WT	Predicted Mutant
Model 1: Clinical and CT – Full Feature Set	WT (n=98)	82	16
	Mutant (n=40)	17	23
Model 2: Clinical and CT – Selected Features Only	WT (n=98)	83	15
	Mutant (n=40)	11	29
Model 3: Radiomics – All Extracted Features	WT (n=98)	77	21
	Mutant (n=40)	21	19
Model 4: Radiomics – Filtered Key Features	WT (n=98)	82	16
	Mutant (n=40)	13	27
Model 5: Combined – Selected CT, Clinical, and Radiomic Features	WT (n=98)	89	9
	Mutant (n=40)	13	27
